# Insurance on Lives—Medical Certificates—Medical Evidence—Remarks on a Recent Trial

**Published:** 1829-01-01

**Authors:** 


### XXXVI.

### MEDICAL JURISPRUDENCE.

#### Insurance of Lives—Medical Certificates—Medical Evidence.

There is a branch of medical jurisprudence which is every day increasing in extent, from the general extent of insurances on lives, and in which, we are sorry to say, the medical character is daily exposed to obloquy, and suspicion of its integrity—not so much, perhaps, from want of principle in individuals, as want of reflection on the duty they are called upon to perform. A policy of insurance cannot be effected without a medical certificate, as to the constitution and state of health of the individual, and the ordinary medical attendant is, of course, referred to by the party proposing to insure. We acknowledge that the physician or surgeon thus appealed to is placed in an awkward situation. If he reveals any defect in the constitution, he risks the rejection of the proposal at the insurance office, and, perhaps, some umbrage on the part of his former patient;—but if, on the other hand, he conceals any flaw in the said life, he not only deviates from the right line of moral rectitude, and thus exposes himself to the loss of character for veracity—but he exposes the surviving family of the party insured, to disastrous law-suits and forfeiture of the policy! All concealments of disorders or tendencies to disease—all misrepresentations of sound constitution, vitiate the policy, according to law and justice, and shake the foundations of those reciprocal advantages which the insuring and insured parties confer and receive. We have daily opportunities of witnessing the unenvied situation in which medical men place themselves by inconsiderate (to give it the mildest term) attestations of sound health, when a minute's examination detects an unsound constitution—too often, actual disease of some important organ! These instances of loose and improper certificates from medical men are now hourly coming before whole companies of respectable and influential members of society, who must and do form, in consequence, a low estimate of the medical character, thus injuring the whole profession. The evil is still farther increased by the litigations which naturally ensue, as effects of these unjustifiable certificates, and the wide range which the exposure of the medical character takes through the medium of the press. We need only refer to a recent trial between Baron Von Lindenau and the Atlas Insurance Company, where a system of medical misrepresentation came out that is quite sickening to peruse. The late Duke of Saxe Gotha was insured in the above office, for three or four thousand pounds, in the year 1824, on the faith of medical attestations, though it turned out that the Duke was even then reduced to a state of idiocy, mutism, and cecity, by an organic disease—a large tumour in the skull, pressing on the brain! Yet the Duke's physicians, Drs. Dorl and Zeigler (shame on such physicians!) certified that they had known the Duke for many years—that his general health was good—that he was merely "*hindered* in the faculty of speech"—and had an affection of the left eye! The evidence of the Duke's physicians and servants went to matters of fact—and we have no hesitation in saying, that these facts were concealed or misrepresented. The evidence of Mr. Green touched matters of opinion—and these matters of opinion we shall quote.

"Mr. *Green* examined.—I am a surgeon in London, and lecturer at St. Thomas's hospital. I have attended to the evidence concerning the Duke's health. I am of opinion that the tumour in the skull must, during life, have been in a passive state. I think, from the appearance in the skull, that it must have been formed in very early life. Little change could have taken place after ossification, and the base of the skull is one of the parts earliest ossified. It is the nature of organic conformations like that to produce ailments which continue to increase. When such tumours exist in a state that may be termed morbid the ailments increase. Supposing the dissection had not taken place, and knowing only what I have heard to-day of the state of the duke's health before he went to Italy, I should say there was no symptom of an organic disease. The spasms which followed the tertian fever, and even spasms more violent, might have arisen from causes quite unconnected with the brain. The attacks, gradually diminishing and ultimately ceasing, would confirm me in my opinion that they did not proceed from the brain. From the cure of the derangement of his digestive organs at the baths of Marienbad, I should still conclude the brain was healthy. After the dissection, supposing a knowledge of the tumors discovered in the brain, knowing the whole course of his illness, and taking into account the care taken of the duke, I should say, that the probability before his death was that he would have lived five years longer. With respect to what has been said of the duke's difficulty of speech, I hardly know what cause to ascribe it to, from the imperfect manner in which it has been described; but I think it was in the mind rather than in the tongue. I am inclined to ascribe it to a want of volition. I do not think that it is to be ascribed to the tumours in the brain. I should not consider that the catarrhal attacks he had passed through would render it less probable that he would live. I have heard the state of the duke's mind described, but do not think it amounts to idiocy, imbecility, or derangement—quite the reverse.

"Cross-examined by Sir *J. Scarlett.*—Do you not consider vigour of mind to be the reverse of imbecility and intelligence of idiocy?—Yes.

"Then you consider the evidence adduced in this case as shewing Duke Frederic to have possessed an intelligent and vigorous mind! I have not said so.

"No, but you say his state was the reverse of imbecile and idiotic; and you allow that vigour of mind and intelligence constitute the reverse of these conditions. When you hear that the duke was 'controlled in his intellect'—that he was watched like a child—that he was so lethargic that he did not move unless desired to do so, and that he, a prince, suffered himself, at his parties, to be turned round and round for the amusement of his guests—do you, I ask, consider these facts as evincing a vigorous intellect?—I have heard the evidence imperfectly, if all these things appear. I heard that he had perception, memory and other mental qualities; that he was fond of music, and of hearing reading.

"Do you think that the symptoms pointed out the existence of disease in the brain?—I do not think so.

"If a patient had had occasional attacks of spasms—if he had been unable to speak for two years—if he had been so indolent as never to move about except when asked to do so—if he was imbecile in his mind—and if, after he became speechless, he was in the habit of frequently putting his hand to his forehead—would you regard these circumstances as indicating any disease of the head?—These symptoms would lead to a *suspicion* of disease there.

"If the patient had an attack, with redness about the face—became affected with paralysis, and died; and if, on opening the head, you found a tumor of the brain, and ten ounces of water effused, would these appearances confirm you in your suspicion, and prove that it had been well-founded?—I cannot give a direct answer to that question. Pressure on the brain, causing loss of speech, would also have produced other symptoms.

"But suppose a case such as I have described?—It is a case I cannot suppose.

"Might not the copious discharge during the catarrhal fever have relieved the brain?—Possibly it might, but these are cases not frequently falling under my observation.

"Might the sudden suppression of such discharge have affected the brain?

—Perhaps it might, by a kind of metastasis.

"What do you mean by that?—That an effusion of a different fluid might have taken place upon the brain from the suppression of the catarrhal discharge.

"Do you think the effusion found on the brain had taken place gradually or suddenly?—Suddenly.

By Lord *Tenterden.*—" If I as a medical man, was asked by an insurance company concerning the state of a man's health who was unwilling to move, who was subject to control upon his intellect, and who had lost his speech, I should not consider myself at liberty to forbear mentioning these circumstances.

"Lord *Tenterden.*—Then there is an end of the cause, for that is the state of the duke described by Dr. Dorl, who signs the certificate sent to the assurance-office.

"*Plaintiff non-suited.*"

We know not what to say to the foregoing evidence. Heaven forbid that we should find fault with a man on account of his opinions, merely because those opinions may differ from our own; yet we think there will be but one opinion throughout the profession respecting the majority of Mr. Green's conclusions—namely, that they were erroneous. Mr. Green's statements shew, in a clear point of view, the bad effects of directing too exclusively (as surgeons often do) their talents and attention to operations, anatomy, and purely surgical diseases, to the neglect (comparatively speaking) of internal pathology—or, in plainer language, the nature, effects, and phenomena of diseases. We hope this short article will awaken our professional brethren to the necessity of guarding the character of themselves, and the faculty at large, from the reproaches which are gathering like a cloud, and threatening to cast a shade on their integrity, as far as regards the granting of medical certificates for the insurance of lives.

---

